# Supplementary material for: Novel Zebrafish Patient-Derived Tumor Xenograft Methodology for Evaluating Efficacy of Immune-Stimulating BCG Therapy in Urinary Bladder Cancer
Source: Cells. 2023 Feb 3;12(3):508. doi: 10.3390/cells12030508 (PMC9914090; doi:10.3390/cells12030508)
Supplement: Supplementary file 1 [file cells-12-00508-s001.zip › cells-2077419-supplementary.pdf]

Supplemental information

Supplemental Table S1: Calculated BCG:cell ratios for each concentration:

**BCG concentration / ml**	**Cell number/nl**	**BCG:cell ratio**
3×10^7	~300 cells	~1:10
4.5×10^7		~1:7.5
6×10^7		~1:5
1.2×10^8		~1:2.5
